# Spatial Alignment of Organoids Tracking Subclonal Chemotherapy Resistance in Pancreatic and Ampullary Cancer

**DOI:** 10.3390/bioengineering10010091

**Published:** 2023-01-10

**Authors:** Md Shahadat Hossan, Ethan Samuel Lin, Eleanor Riedl, Austin Stram, Eric Mehlhaff, Luke Koeppel, Jamie Warner, Inem Uko, Lori Mankowski Gettle, Sam Lubner, Stephanie M. McGregor, Wei Zhang, William Murphy, Jeremy D. Kratz

**Affiliations:** 1 Division of Hematology, Medical Oncology and Palliative Care, Department of Medicine, School of Medicine and Public Health, University of Wisconsin, Madison, WI 53705, USA; 2 Department of Radiology, School of Medicine and Public Health, University of Wisconsin, Madison, WI 53792, USA; 3 University of Wisconsin Carbone Cancer Center, School of Medicine and Public Health, University of Wisconsin, 600 Highland Ave., Madison, WI 53705, USA; 4 William S. Middleton Veterans Administration Health System, Madison, WI 53705, USA; 5 Department of Pathology and Laboratory Medicine, School of Medicine and Public Health, University of Wisconsin, Madison, WI 53705, USA; 6 Department of Biomedical Engineering, University of Wisconsin, Madison, WI 53706, USA; 7 Department of Orthopedics and Rehabilitation, University of Wisconsin, Madison, WI 53705, USA; 8 Department of Materials Science and Engineering, University of Wisconsin, Madison, WI 53706, USA; 9 Center for Human Genomics and Precision Medicine, University of Wisconsin, Madison, WI 53705, USA

**Keywords:** organoids, therapeutic response, cancer heterogeneity, pancreatic cancer, ampullary cancer, apoptosis, necrosis

## Abstract

Pancreatic and ampullary cancers remain highly morbid diseases for which accurate clinical predictions are needed for precise therapeutic predictions. Patient-derived cancer organoids have been widely adopted; however, prior work has focused on well-level therapeutic sensitivity. To characterize individual oligoclonal units of therapeutic response, we introduce a low-volume screening assay, including an automated alignment algorithm. The oligoclonal growth response was compared against validated markers of response, including well-level viability and markers of single-cell viability. Line-specific sensitivities were compared with clinical outcomes. Automated alignment algorithms were generated to match organoids across time using coordinates across a single projection of Z-stacked images. After screening for baseline size (50 μm) and circularity (>0.4), the match efficiency was found to be optimized by accepting the diffusion thresholded with the root mean standard deviation of 75 μm. Validated well-level viability showed a limited correlation with the mean organoid size (R = 0.408), and a normalized growth assayed by normalized changes in area (R = 0.474) and area (R = 0.486). Subclonal populations were defined by both residual growth and the failure to induce apoptosis and necrosis. For a culture with clinical resistance to gemcitabine and nab-paclitaxel, while a therapeutic challenge induced a robust effect in inhibiting cell growth (GΔ = 1.53), residual oligoclonal populations were able to limit the effect on the ability to induce apoptosis (GΔ = 0.52) and cell necrosis (GΔ = 1.07). Bioengineered approaches are feasible to capture oligoclonal heterogeneity in organotypic cultures, integrating ongoing efforts for utilizing organoids across cancer types as integral biomarkers and in novel therapeutic development.

## 1. Introduction

Pancreatic and ampullary cancers are aggressive malignancies projected to become leading causes of cancer-related mortality by 2030 [1]. The most aggressive disease histology of adenocarcinoma exhibits hallmarks of early metastases and therapeutic resistance, with 5-year survival estimated at 10% [2]. The current standard of care therapies include combination chemotherapy with FOLFIRINOX (FFX; 5-fluorouracil, irinotecan, and oxaliplatin) [3] and the combination of gemcitabine and nab-paclitaxel (gem/nab-pac) [4]. To date, there are no prospective clinical trials to differentiate sensitivities between these therapeutic regimens to personalize therapeutic strategies. There remains a significant opportunity to improve precision strategies for these cancers through screening applications, as well as to develop novel biomarkers for clinical prediction.

Several barriers have been discovered in the development of biomarkers predictive of effective systemic therapies. The tumor microenvironment, including a highly immunosuppressive and dense desmoplastic stroma, remains a formidable barrier [5]. As such, therapeutic strategies must maintain large therapeutic indices to achieve a response; however, they must also consider the high degree of inter- and intra-tumor heterogeneity within pancreatic ductal adenocarcinoma (PDAC) [6,7]. Additionally, cancer stem cells (CSCs) in pancreatic cancer are highly plastic in mediating adaptive resistance to both chemotherapy and radiotherapy [8,9]. Characterizing subclonal resistance cannot be achieved clinically due to high costs for serial biopsy and few identified precision targets in these cancer types.

Historical models of pancreatic and ampullary cancers include two-dimensional (2D) cell cultures or patient-derived xenograft (PDX) models, while patient-derived cancer organoids (PCOs) have significantly grown in popularity over the past decade [10,11,12]. Unfortunately, traditional 2D cell cultures do not fully recapitulate the complexities of cancer biology and harbor inconsistencies in culture development with the selection of subclones for enhanced growth and adhesion. PDX models have been well established for in vivo therapeutic investigations; however, these models incur added financial costs associated with maintaining animal colonies. These costs increase rapidly, especially when considering the screening needed for combination therapy across the breadth of therapeutic development. Comprehensive next-generation sequencing has shown that molecular profiles of patient-derived organotypic cultures preserve concordance with primary tumor and established PDX models, with signatures predictive of clinical response [13,14,15].

Organotypic cultures in a 3D matrix have enabled the evaluation of disease biology in an efficient, cost-effective, and high-throughput manner [16]. PCOs can be developed from a variety of clinical samples (e.g., biopsy, surgical resection, or malignant effusion). These models are established quickly, at low cost, with improved success in comparison to patient-derived xenografts [17,18,19]. These cultures maintain the features of the cancers from which they were derived, including genetic alterations, metabolism, and drug response [10,20,21,22,23,24,25]. Organoids from patient-derived tumors maintain the cell–cell communication and the 3D architecture needed to accurately assess the individualized drug response [10,24,25,26].

Accurate models for the prediction of therapeutic response must consider the contributions of oligoclonal populations. Cancer heterogeneity in pancreatic models is expanded when transitioning cultures from 2D to 3D using spheroid or patient-derived organotypic applications [27]. The challenge remains that few phenotypic screening assays consider the contributions of the oligoclonal elements within a given culture. Most assays rely on a well-level viability response using dosing extensions beyond physiologic values [11,12]. Unique techniques for response have included the use of optical metabolic imaging and high content fluorescence imaging to train a neural-network model, both with applications in label-free drug response prediction [28,29].

To date, there are no tools that utilize subclonal heterogeneity for the prediction of distinct clinically resistant populations. While a majority of pathologic drivers are preserved, autopsy series from patients with definitive surgical management highlighted the importance of subclonal populations with the selection of resistant populations at the time of disease recurrence [30]. While cancer evolution can be tracked using molecular profiling, no clinical tools exist to track this resistance or predict which subclonal population will confer resistance for an individual patient. Furthermore, resistance is mediated at the level of both subclonal DNA alterations and complex epigenetic and transcriptional regulation [31,32]. There remains a critical unmet need to map such subclonal populations of cancer in the oligoclonal resistant population within a given tumor. PCOs are emerging as an important model systemic, given their role in expanding subclonal populations and longitudinal applications to understanding therapeutic resistance.

In this study, we generate an automated alignment algorithm to track the population-level organoid response in pancreatic and ampullary PCO models. The primary objective was to characterize the individual organoid response by tracking this across hundreds of individual organoids. The study aimed to establish thresholds for the stochastic diffusion of organoids in commercially available matrices. Additionally, line-specific sensitivities were established, with varied sensitivities against single agents and a combination standard of care chemotherapy. We hypothesized that subclonal populations exist with the failure to induce apoptosis and necrosis. When adapted to screen level applications, well-level viability was not predictive of organoid size or growth, consistent with the subclonal resistance observed within cultures. While both well-level and growth have served as a surrogates for response, the failure to induce an irreversible intraorganoid response by apoptosis and necrosis remains a formidable barrier for successful therapeutic development.

## 2. Materials and Methods

### 2.1. Tissue Processing

All studies were completed following institutional review board (IRB) approval, with consent obtained from subjects prior to tissue acquisition from the UW Translational Science BioCore (UW IRB#2016-0934). Briefly, tissue was obtained from needle biopsy and primary surgical resection, with different sampling sites (Table S1). The sample was placed in chelation buffer for at least 30 min. Digestion was performed in base media (Table S2) with collagenase 1 mg/mL (Sigma, St. Louis, MO, USA), papain 0.4 U/mL (Worthington, NJ, USA), and ROCK inhibitor Y-27632 10 µM (Medchemexpress, Princeton, NJ, USA). The tissue was grossly dissected for use in the suspension using a razor blade and digested for 30 min at 37 °C with intermittent shaking and two mechanical disruptions using a gentleMACS Dissociator (Miltenyi Biotec, Bergish Gladbach, Germany). Malignant fluids (pleural effusions) were initially pelleted and separated using a Ficoll preparation (Cytiva, Uppsala, Sweden). The tissue suspension was centrifuged at 700× *g* at 4 °C for 5 min and resuspended in the base media. The PCO suspensions were immediately mixed at a 1:1 ratio with Cultrex Growth Factor Reduced Basement Membrane, Type 2, Select (R&DSystems, Minneapolis, MN, USA). Droplet suspensions were plated and set for 3–5 min at 37 °C, then inverted for at least 30 min to solidify the matrix and avoid direct contact of PCOs with the plastic interface. The plated cultures were covered with feeding medium and incubated at 37 °C in 5% CO_2_, with the regular medium replaced every 72 h. The media formulas were adapted from existing protocols optimized for pancreatic and hepatobiliary applications [29]. For selective WNT activation in serum free conditions, the next generation WNT surrogate peptide was included at 500 pm (Immunoprecise, Utrecht, Netherlands) for selective activation [29,33].

### 2.2. Response Assay Preparation

The cultures were expanded for multiple passages (at least 3) prior to conducting the therapeutic assays. For passaging, base media was added to the wells to solubilize the matrix using mechanical disruption. Cell suspensions were centrifuged at 700× *g* at 4 °C for 5 min. Organoid pellets were resuspended in the base media in a 1:1 ratio with the matrix prior to plating. Tissue culture-treated 24-well plates (VWR, Randor, PA, USA) were used for traditional passaging and growth characterization (as above). Low volume assays were performed by plating 500 cells/μL. All cells were counted using a Countess II (Invitrogen, Bothell, WA, USA) using a 1:1 cell suspension mixture with trypan blue 0.4% (VWR, Solon, OH, USA). Low volume assays were performed in a 96-well low-volume angiogenesis plate (Ibidi, Bavira, Germany), with a matrix volume of 10 µL per well. Due to improved hydrostatic pressure, cultures were inverted immediately after plating in the 96-well format. All treatment and response assays were initiated 24 h after culture plating.

### 2.3. Organoid Histology

After culture expansion, at least 16 confluent wells were selected for fixation. The media was replaced with 2% paraformaldehyde (PFA) (Sigma), without matrix disruption, for 15 min. Then, the matrix was mechanically disrupted with a collection of PFA-based organoid suspensions. The fixed cells were centrifuged 1000× *g* for 5 min at 4 °C. The supernatant was aspirated and resuspended in 50% EtOH (Decon, Prussia, PA, USA). The organoids were repeatedly centrifuged and resuspended with increasing amounts of EtOH for dehydration (70%, 80%, 95%, and 100%). Dehydrated organoid suspensions were maintained at 4 °C and provided to the University of Wisconsin Carbone Cancer Center (UWCCC) Experimental Animal Pathology Lab for block preparation, slide processing, and H&E staining. All slide imaging was performed with a 40× objective with an Eclipse E200 (Nikon, Tokyo, Japan), and images were prepared using an NIS-Elements D (Nikon).

### 2.4. Chemotherapy Response Assessment

At the time of assay, baseline images were collected prior to pharmacologic agent preparation. Dosing was performed using a physiologic Cmax of 5-fluorouracil (5-FU) 10 μM [34], oxaliplatin 5 μM [35], SN-38 1.5 nM [36], gemcitabine 50 μM [37], and nab-paclitaxel 10 μM [38]. All treatments were changed daily, with continuous therapy of nab-paclitaxel (72 h), while 5-FU, oxaliplatin, and SN-38 were provided for 48 h, and gemcitabine was used for 24 h only to model the early clearance pharmacokinetics [39]. The media was replaced every 24 h over the duration of the experiment in all wells, including control. The pharmacologic agents were received from the UWCCC Pharmacy, including 5-FU (Fresenius, Lake Zurich, IL, USA), gemcitabine (Hospira, Lake Forest, IL, USA), oxaliplatin (Hospira), and nab-paclitaxel (Twi, Paramus, NJ, USA). Additional agents were prepared from stock powders, including SN-38 (MedChemExpress, Princeton, NJ, USA). All DMSO concentrations were maintained <0.1% *v*/*v* across all experimental groups. The effect size was measured using Glass’s Delta (GΔ) to account for the difference between the treatment and control group as normalized to the standard deviation of the control population. An effect size for the normalized Δ diameter was considered with intermediate response, with threshold >1.25, based on prior reports [40,41].

### 2.5. Viability Staining

After 72 h, brightfield imaging was performed prior to staining. The media was aspirated with the addition of a staining solution, including CD117 Violet Blue 0.5% *v*/*v* (BioLegend, San Diego, CA, USA), ToPro3 0.3% *v*/*v* (Invitrogen), and Caspase3/7 2.5 µM (Sartorius, Gottingen, Germany), in the base media. To validate the index of the diameter, PCOs were stained with Hoechst 33342 13 µM (Invitrogen, Eugene, OR, USA). All staining was performed for 2 h at 37 °C. Once incubation was complete, the staining solution was aspirated off, and the wells were washed twice with 1× PBS (Gibco, Grand Island, NY, USA) prior to imaging.

### 2.6. Therapeutic Screening

PDAC PCOs were plated in a 96-well assay (as above), including 5000 cells in a 10 µL matrix. A custom library of 80 lead therapeutic agents was identified from ongoing phase I/II clinical trials in development. These agents were prepared per the manufacturing guidance (MedChemExpress, Princeton, NJ, USA) and dosed at physiologic values up to 5 μM (Table S3). Small molecules were added using Echo 550 (Beckman Coulter, Indianapolis, IN, USA) for low-volume liquid handling in collaboration with the UWCCC Drug Development Core. No pharmacologic adjustments or media replacements were performed over the duration of therapeutic screening. Following drug incubation, cell viability was measured by adding 3D Cell Titer-Glo luminescence 33.3% *v*/*v* (Promega, Madison, WI, USA). After adding the reagent, the plate was shaken for 5 min, incubated for 25 min at room temperature in the dark, and the luminescence was measured using a Gen5 plate reader (Biotek, Santa Clara, CA, USA).

### 2.7. Brightfield Imaging

Brightfield imaging was performed at 0 h and 72 h using Cytation5 (Biotek) with a 4× objective with a 5 field Z-stack (±2 fields of view ×100 µm from the autofocused plane). Subsequent image preprocessing was performed to control background signal intensity to render a single Z-projection for analysis. Individual objects were defined based on an automated contrast threshold between the edges of regions of interest and the background of the plate. Maximum diameter, area, x-coordinate, y-coordinate, and circularity were exported. Objects with a diameter between 50 µm and 750 µm were included in the analysis. The normalized change in diameter was compared using the difference between 72 h and 0 h, normalized to the 0 h value. This was repeated for individual objects across 3 experimental replicates and 3 technical replicates for all chemotherapy treatment studies.

### 2.8. Fluorescence Imaging

After staining, fluorescence imaging was performed using identical fields of view, using the autofocus, as defined from brightfield imaging. The images underwent identical Z-projections and image pre-processing per each replicate. The excitation parameters were held constant between technical replicates; however, there were minor variations in exposure timing to control for minor fluctuations in day-specific fluorophore sensitivity (Table S4). All values were compared relative to the untreated media control across at least 3 experimental replicates and 3 technical replicates for all chemotherapy treatment studies.

### 2.9. Semi-Automated Matching of Objects

Object-level metrics were collected and processed within a program hosted in Microsoft Excel. Circularity (Equation (1)) was the first exclusionary criteria measured as continuous between 0 and 1, defined as:

(1) $$\mathrm{Circularity}=\frac{{\mathrm{Perimeter}}^{2}}{4\pi \times \mathrm{Area}}$$

Utilizing the x and y coordinates a nearest neighbor matrix (NNM) was generated by assessing the Euclidean distance, or root mean standard deviation (RMSD), between each object in the units of μm:

(2) $$\mathrm{RMSD}=\sqrt{{{(y}_{2}{-y}_{1})}^{2}{+(x}_{2}{-x}_{1}{)}^{2}}$$

The NNM was subsequently converted into a binary validation matrix (BVM), in which distances below or equal to a user-defined value of Euclidean distance were coded into values [0, 1] representing true matches (1) or false matches (0). Objects with multi-mapping behavior were excluded from all analyses.

### 2.10. Optimized Selection Criterion

The assumption was made that successful matches of objects that pass both of circularity and RMSD operators were single organoids. The function of variance (K) was defined using the equation:

(3) $$K={\left(\sum \left|M-X_{replicate}\right|\right)}^{2}$$

where M is the median of the aggregate percentage of the objects matched at a given circularity index, and X is the percentage of objects matched.

## 3. Results

### 3.1. Low Volume Organoid Screening Supports Organoid Development

**Figure 1:**
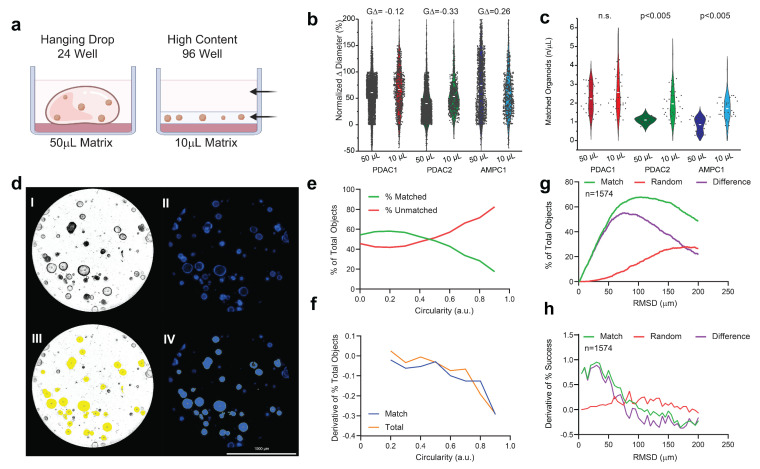
Evaluation of low−volume organotypic screening. (**a**) Design of traditional hanging drop format of the 50 µL in 24−well plates versus the adapted high−content design of the 10 µL matrix in low−volume 96-well screening. (**b**) Violin plots of the normalized change in diameter for three established PCO organoid lines with effect sizes (GΔ) normalized to the traditional hanging drop. (**c**) Violin plots of the number of organoids established per µL of the matrix with corresponding Student t−testing for each pairwise set. (**d**) AMPC organoids imaged in media-only, conditioned and assayed using an established marker for viability (Hoescht) at 72 h; scale bar is 1000 μm, including (**I**) the brightfield image of AMPC1, (**II**) image of Hoescht-stained AMPC1, (**III**) object selection through Gen5 on the brightfield image, and (**IV**) object selection through Gen5 using only Hoescht staining. (**e**) The percentage of the total object population, matched and unmatched, across the continuous variable of the circularity index. (**f**) First derivative of change in objects for matched and total selected conditions across the circularity index. (**g**) Optimal RMSD is shown by the local maximum of the difference (purple) between the percentage of successful matches between two true inputs (green) and random assorted images (red). (**h**) First derivative of all three inputs to evaluate alignment, where match rate is surpassed by random rate.

Across all three independent cultures, PCOs plated in a 96-well low-volume format showed a similar normalized Δ diameter versus the expansion cultures, as assayed at 72 h (Figure 1a,) without any meaningful difference in population effect size (GΔ). When assayed with identical concentrations (500 cells/μL), the PDAC2 and AMPC cultures showed improved object matched at +186% (*p* < 0.005) and +224% (*p* < 0.005), respectively (Figure 1c).

### 3.2. Circularity and Euclidean Distant Thresholds for Accurate Organoid Alignment

The brightfield imaging was predictive of Hoescht staining as assayed by area (R = 0.991, Figure 1d and Figure S1). Circularity was considered as a continuous variable across increments of 0.1. The percentage of matched objects was surpassed by the percentage of unmatched objects beyond 0.4 (Figure 1e,f). The second selection operator of Euclidean distance (RMSD) was considered as a continuous variable from 1 µm and 200 µm. At 75 µm, there was maximum match success of 63.3%, when evaluated across 8 experimental sets (Figure 1g). Importantly, there was an increase in false matches using random assessment when exceeding RMSD > 75 μm, consistent with the first derivative in considering the difference between matched and random imaging alignment (Figure 1g,h).

### 3.3. Well-Level Viability Has Limited Predictive Values against Organoid Growth

**Figure 2:**
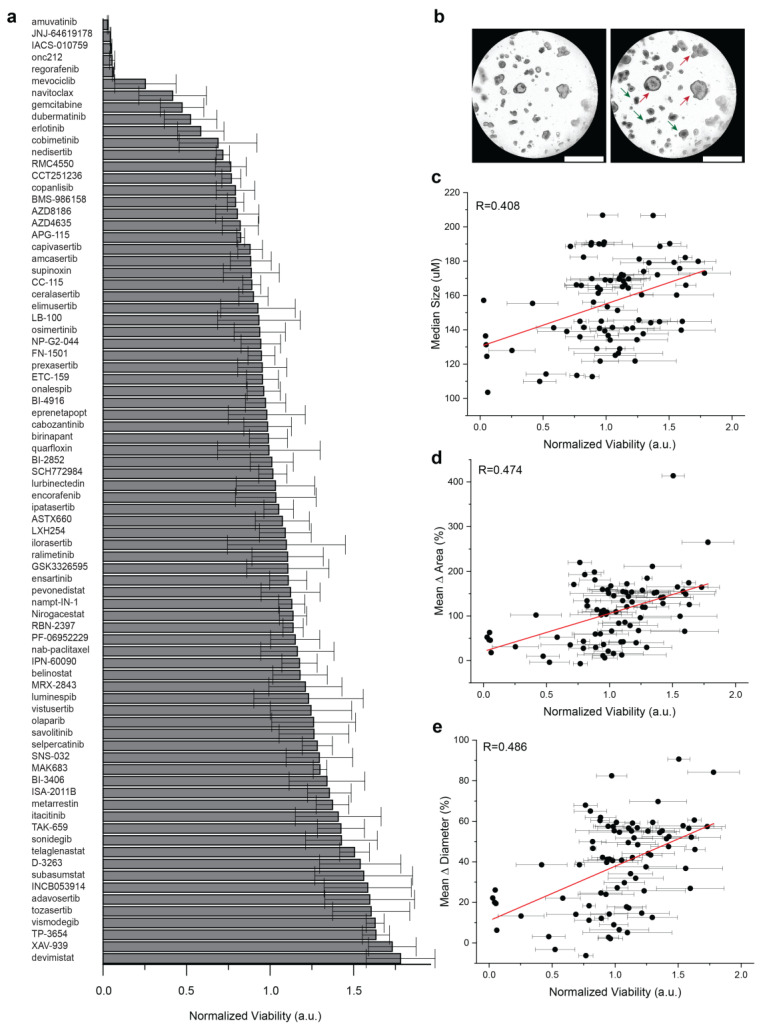
Comparison of population-based organoid response and well-level viability. (**a**) Bar chart of therapeutic response in PDAC2 after 72 h, normalized to control viability across 80 agents used in early phase clinical development. Error bars represent standard deviation for normalized viability across four technical replicates. (**b**) Representative brightfield imaging at 0 h and 72 h, showing evidence of subclonal response heterogeneity after treatment with 1 μM navitoclax, including organoids with phenotypic resistance (red) and response (green). (**c**,**d**) Dot plots defined to compare normalized well-viability from screening (**a**) against (**c**) median organoid size alone at 72 h; (**d**) mean organoid Δ in area normalized to start of treatment; (**e**) mean organoid Δ in diameter normalized to start of treatment. Error bars represent standard deviations for normalized viability across four technical replicates. The Pearson correlation coefficient (R) is shown with data generated, including the evaluation of n = 6381 organoids.

To compare well-level viability to organoid growth, a medium throughput screening was performed in PDAC2 culture using 80 agents used in early phase clinical trials (Figure 2a). Across 4 technical replicates, a wide range of therapeutic responses was observed for single agents, with a fixed dose up to 5 μM (Table S3). Importantly, subclonal resistance was readily apparent in cultures reported with representative treatment of the single agent navitoclax, including a normalized viability < 0.5; however, clear organoid growth was observed in the culture (Figure 2b). Limited correlations were observed for well-level normalized viability and median organoid size (R = 0.408, Figure 2c), mean Δ area (R = 0.474, Figure 2d), and mean Δ diameter (R = 0.486, Figure 2e).

### 3.4. Organoid Sensitivity Can Be Tracked by Automated Population Growth

**Figure 3:**
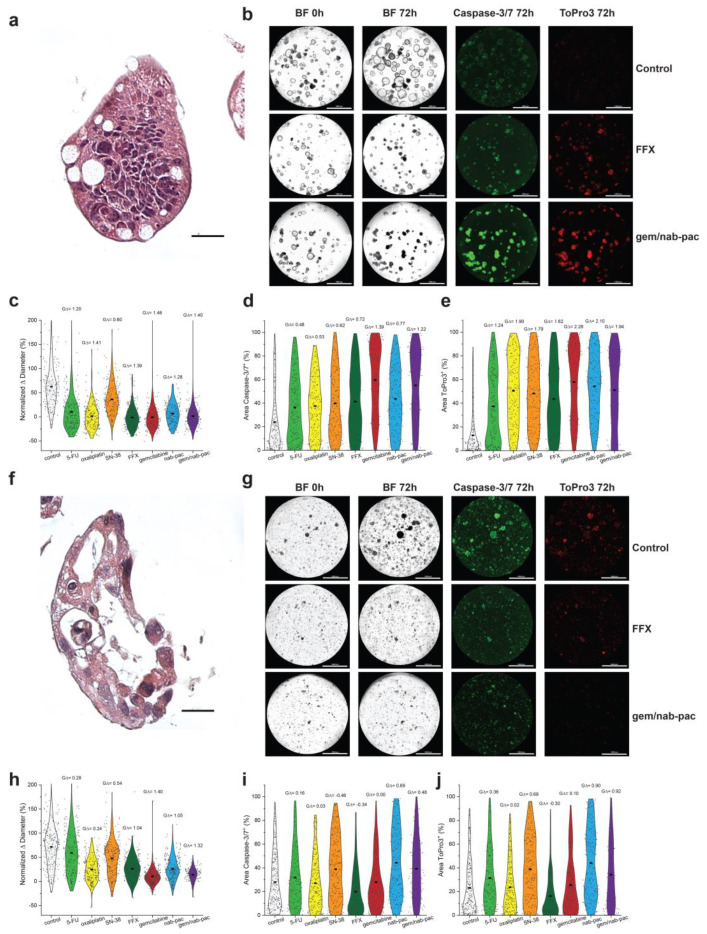
Therapeutic response assessment in organoids with a validated viability marker. (**a**) H&E staining of AMPC1, with corresponding nuclear atypia, ahigh nuclear to cytoplasmic ratio, and nuclear atypia. (**b**) Images of AMPC1 organoids by treatment type, including media control (top row), FFX (second row), and gem/nab-pac (third row), with brightfield microscopy at 0 h and 72 h, and fluorescence microscopy for caspase-3/7 (apoptosis) and ToPro3 (necrosis) at 72 h. (**c**) Violin plot of normalized % change in diameter of AMPC1 when treated with chemotherapies, as well as their respective effect sizes. (**d**) Violin plot of % of area positive for caspase-3/7 of AMPC1 when treated with chemotherapies, along with their respective effect sizes. (**e**) Violin plot of % of area positive for necrosis marker ToPro3 for AMPC1 when treated with chemotherapies, as well as their effect sizes. (**f**) H&E staining of PDAC1 organoids showing nuclear atypia and a high N/C ratio. (**g**) Images of PDAC1 organoids, including media control (top row), FFX (second row), and gem/nab-pac (third row) with brightfield microscopy at 0 h and 72 h, and fluorescence microscopy for caspase-3/7 (apoptosis) and ToPro3 (necrosis) at 72 h. (**h**) Violin plot of normalized % change in diameter for PDAC1 when treated with chemotherapies, along with their effect sizes. (**i**) Violin plot of % of area positive for caspase-3/7 of PDAC1 when treated with chemotherapies, as well as their respective effect sizes. (**j**) Violin plot of % area positive for necrosis marker ToPro3 for PDAC1 when treated with chemotherapies, along with their respective effect sizes. Scale bars are 20 μm for IHC images (**a**,**f**) and 1000 μm for well-level microscopy (**b**,**g**).

AMPC1 stained with H&E showed ongoing nuclear atypia and high nuclear to cytoplasm ratios, as well as mucin production consistent with advanced cancer (Figure 3a,f). Given the clinical uncertainty of chemotherapy in ampullary cancer, AMPC1 was treated with a panel of different chemotherapies. When tracked for growth, AMPC1 was found to have similar sensitivity to gem/nab-pac (mean growth +1%, GΔ = 1.40) and FFX (mean growth +2%, GΔ = 1.39, Figure 3b,c) and harbored exquisite sensitivity to gem (mean growth +0%, GΔ = 1.46); however, it was found to be resistant to single agent SN-38 (GΔ = 0.60).

PDAC1 was found to have nuclear atypia and apoptotic bodies consistent with an aggressive clinical presentation (Figure 3f). PDAC1 exhibited sensitivity to gem/nab-paclitaxel chemotherapy (mean growth +10%, GΔ = 1.32); however, a significant population showed resistance to FFX (mean growth +26%, GΔ = 1.04; Figure 3g,h). Persistent growth was observed across mean values for all single agents, as well as the combination chemotherapy regimens. This aggressive disease histology was consistent with a clinical history that included mortality on the order of few days from subsequent hepatic failure.

### 3.5. Organoid Sensitivity Can Be Tracked for Markers of Induced Apoptosis and Necrosis

Consistent with chemotherapy achieving growth arrest, AMPC1 transitioned from markers of apoptosis (Figure 3d) to markers of necrosis (Figure 3e). The response by induced apoptosis was increased for gem/nab-pac (GΔ = 1.22), largely driven by gemcitabine (GΔ = 1.39), and improved when compared to combination FFX (GΔ = 0.72). The response for necrosis was also increased for gem/nab-pac (GΔ = 1.94), largely driven by gemcitabine (GΔ = 2.28), and improved when compared to combination FFX (GΔ = 1.62). When combined with the growth data, these results highlight the differential sensitivity to chemotherapy containing gemcitabine. PDAC1 showed persistent growth across populations, and the contributions for induced apoptosis and necrosis were limited (Figure 3g,i,j). Staining for markers of induced apoptosis showed limited responses for FFX (GΔ = 0.34) and gem/nab-pac (GΔ = 0.48; Figure 3g,i). PDAC1 exhibited a limited response to necrosis when treated with FFX (GΔ = −0.30) and gem/nab-pac (GΔ = 0.92).

### 3.6. Characterizing Multiplex Pancreatic Organoid Response with Clinical Resistance

**Figure 4:**
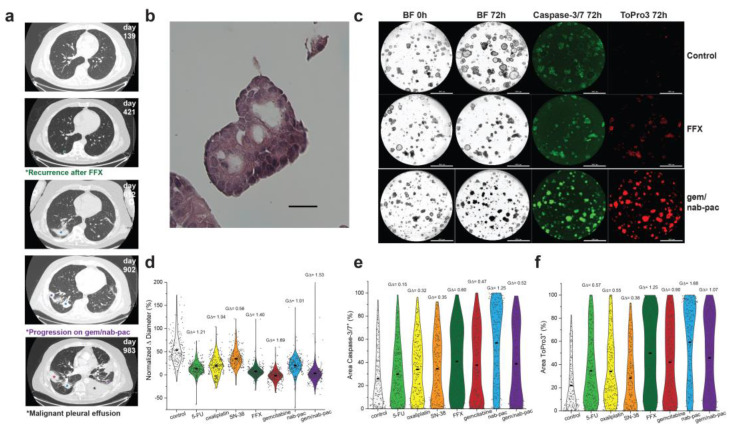
Therapeutic response assessment of clinical resistance. (**a**) Clinical course of PDAC2 including serial CT thorax showing recurrence after neoadjuvant FFX (green*, day 421), pulmonary disease progression (violet*, day 902), and the development of malignant pleural effusion (black*, day 983), with a corresponding site of prior irradiation (blue*). (**b**) H&E staining of PDAC2 organoids displaying typical cancer phenotypes, such as nuclear atypia and a high N/C ratio; scale bar is 20 μm. (**c**) Images of PDAC2 organoids by treatment type, including media control (top row), FFX (second row), and gem/nab-pac (third row), with brightfield microscopy at 0 h and 72 h, and fluorescence microscopy for caspase-3/7 (apoptosis) and ToPro3 (necrosis) at 72 h. The third row shows gem/nab-pac treatment; scalebar is 1000 μm. (**d**) Violin plot of normalized % change in diameter of PDAC2 organoids treated with chemotherapies and their respective effect sizes. (**e**) Violin plot of % of area positive of caspase—3/7 for PDAC2, when treated with chemotherapies, and their corresponding effect sizes. (**f**) Violin plot of % of area positive of ToPro3 for PDAC2 when treated with chemotherapies and their respective effect sizes.

PDAC2 was produced from malignant effusion generated after recurrence with neoadjuvant FFX and disease progression with combination gem/nab-pac (Figure 4a). The expanded organoid culture showed preserved nuclear atypia at early passage (Figure 4b). Here, PDAC2 showed growth arrest with gem (mean growth +2%, GΔ = 1.69), and gem/nab-pac (mean growth +3%, GΔ = 1.53), as well as a response to FFX (mean growth +7%, GΔ = 1.41; Figure 4c,d).

Despite growth arrest, a mixed response was noted for markers of organoid viability (Figure 4c,e,f). A limited increase in the markers of apoptosis was seen for FFX (GΔ = 0.60), gem (GΔ = 0.47), and gem/nab-pac (GΔ = 0.52, Figure 4e). While an increased response was noted for necrosis, including FFX (GΔ = 1.25), gem (GΔ = 0.90), and gem/nab-pac (GΔ = 1.07, Figure 4f), remnant populations remained without necrosis.

### 3.7. Multiplex Analysis of Individual Organoids Captures Growth and Viability

**Figure 5:**
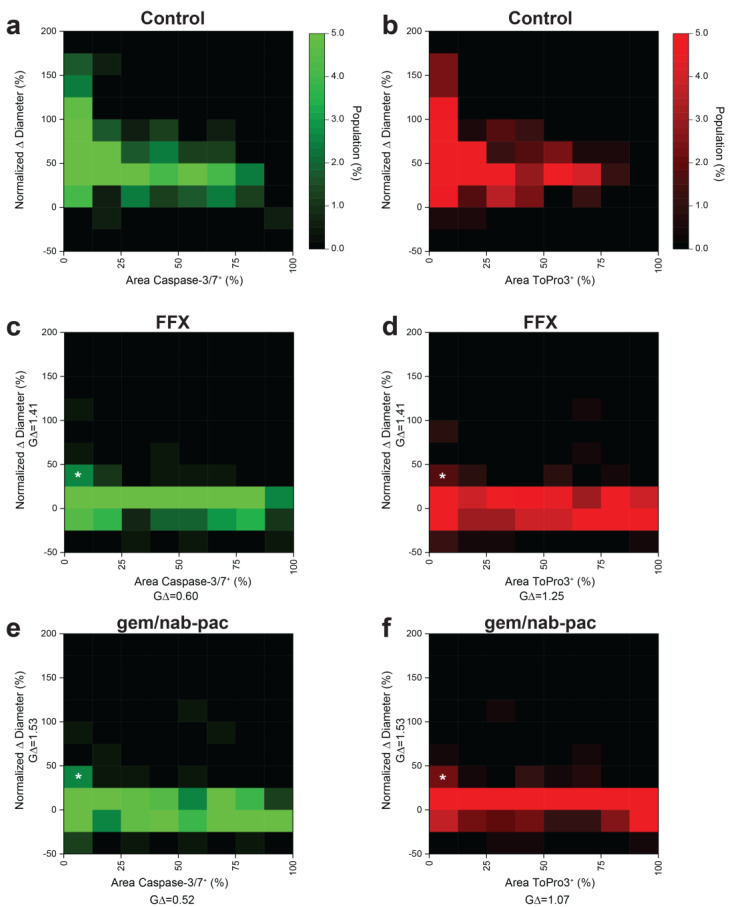
Multidimensional analysis of resistance PDAC organoids with residual markers of viability. (**a**) Heat map showing the population distribution of PDAC2 control organoids, including normalized Δ diameter, plotted against (**a**) % area positive for caspase−3/7 and (**b**) % area positive for ToPro3. (**c**,**d**) Heat map showing the population distribution of PDAC2 after 72 h FFX treatment, with normalized Δ diameter, plotted against (**c**) % area positive for caspase−3/7 and (**d**)% area positive for ToPro3. (**e,f**) Heat map showing the population distribution of PDAC2 after 72h gem/nab-pac treatment with normalized Δ diameter plotted against (**e**) % area positive for caspase−3/7 and (**f**) % area positive for ToPro3. (**c**–**f**) Subclonal populations after treatment with residual growth [+25%, +50%] and <12.5% area positive for viability markers shown (white*).

Given prior disease progression in PDAC2, a dedicated analysis was performed to consider the contributions of growth and viability using multiplex heatmapping (Figure 5). Growth was increased for populations with decreased staining for apoptosis (Figure 5a) and necrosis (Figure 5b) in control populations. As noted previously, growth arrest was seen across the global population with the treatment of FFX (Figure 5c,d) and gem/nab-pac (Figure 5e,f). This subgroup analysis provided remnant visualization of the subclonal populations, with both persistent growth and failure to induced change in the viability by apoptosis and necrosis (Figure 5c–f).

## 4. Discussion

There remains a critical unmet need for novel therapeutic development in PDAC and ampullary cancers with opportunities to interface bioinformed models with bioengineered tools. Here, we present a bioengineered tool to automate the characterization of subclonal populations in a response assessment. Despite advancements in comprehensive and single-cell molecular profiling, understanding the clonal dynamics of the therapeutic response remains a formidable clinical challenge, largely due to the invasive nature of repeat tissue sampling. This study presents a low-volume matrix screening assay to characterize the response to map changes in organoid growth with validated markers of viability, including induced apoptosis and necrosis. This is the first report of characterizing individual growth and validated viability markers across panels of hundreds of individual organoid units. These assays have broad application for investigators using organotypic cultures for considering the contributions of subclonal resistance across cancer biology.

The development of low-volume screening assays can be widely adapted to consider the next-generation of cancer organoids [42,43]. This is particularly important in considering how contributions of the matrix and the tissue microenvironment foster the failure of therapeutic delivery and changes in cancer biology to harbor resistance populations. Recent work by Osuna de la Peña et al. revealed that changes in pancreatic cancer biology can be achieved using peptide amphiphiles coupled with custom extracellular matrix components [44], harboring therapeutic resistant populations with improved biophysical material properties. This feature is of critical importance when consider the hallmarks of PDAC, including tissue-specific density in the desmoplastic stroma. Importantly, this differs significantly from the technologies suggested by Hirokawa et al. that expand endoluminal cancers in low viscosity states [45]. The next frontier of personalized medicine must consider models that capture both the molecular features of cancer biology and the cancer-specific local microenvironment.

These technologies can be considered across multichannel fluorescence in multiplex assays for both markers of viability and hallmarks of resistance (i.e., cancer stem cells). However, such response assessments must also consider how staining may vary as a function of overall organoid size. It was recently reported that baseline size and passage number have minimal contributions regarding baseline organoids growth [46]. It is likely that response characterization by growth and staining for viability are interdependent, which highlights the importance of considering the quantitative metrics of cellularity. The predictive values of such technologies require significant prospective validation. It is important to consider that a viability reading on the order of days may not capture all the adaptive features of these highly aggressive cancers. Validation requires both the controlled timing of tissue sampling and a comparison against durable markers of clinical endpoints (e.g., progression-free survival).

This study contributes a technology to characterize subclonal populations by multiplex response classification to extend beyond well-level assays of organoid viability. The future of screening applications in pancreatic and ampullary cancer organoids offers significant opportunities in the context of developing novel therapeutic targets. We present a multiplexed assay to capture resistance that extends beyond phenotypic changes to consider markers of oligoclonal viability. Interfacing assays that capture the heterogeneity of the subclonal response, with longitudinal endpoints, may lend the use of these technologies for improved clinical prediction.

## Data Availability

The data presented in this study are available on request from the corresponding author.

## Supplementary Materials

The following supporting information can be downloaded at: https://www.mdpi.com/article/10.3390/bioengineering10010091/s1, Figure S1: Comparison of individual organoid size; Table S1: Summary of media composition used for pancreatic and ampullary PCOs; Table S2: Summary of clinical parameters for individual organoid cultures; Table S3: Drug screen list; Table S4: Fluorescent microscopy settings

## References

[1] Rahib L., Smith B.D., Aizenberg R., Rosenzweig A.B., Fleshman J.M., Matrisian L.M. (2014). Projecting cancer incidence and deaths to 2030: The unexpected burden of thyroid, liver, and pancreas cancers in the United States. Cancer Res..

[2] Mizrahi J.D., Surana R., Valle J.W., Shroff R.T. (2020). Pancreatic cancer. Lancet.

[3] Conroy T., Desseigne F., Ychou M., Bouché O., Guimbaud R., Bécouarn Y., Adenis A., Raoul J.-L., Gourgou-Bourgade S., de la Fouchardière C. (2011). FOLFIRINOX versus gemcitabine for metastatic pancreatic cancer. N. Engl. J. Med..

[4] Von Hoff D.D., Ervin T., Arena F.P., Chiorean E.G., Infante J., Moore M., Seay T., Tjulandin S.A., Ma W.W., Saleh M.N. (2013). Increased survival in pancreatic cancer with nab-paclitaxel plus gemcitabine. N. Engl. J. Med..

[5] Ho W.J., Jaffee E.M., Zheng L. (2020). The tumour microenvironment in pancreatic cancer—Clinical challenges and opportunities. Nat. Rev. Clin. Oncol..

[6] Cros J., Raffenne J., Couvelard A., Poté N. (2018). Tumor heterogeneity in pancreatic adenocarcinoma. Pathobiology.

[7] Connor A.A., Gallinger S. (2022). Pancreatic cancer evolution and heterogeneity: Integrating omics and clinical data. Nat. Rev. Cancer.

[8] Ayob A.Z., Ramasamy T.S. (2018). Cancer stem cells as key drivers of tumour progression. J. Biomed. Sci..

[9] Shibue T., Weinberg R.A. (2017). EMT, CSCs, and drug resistance: The mechanistic link and clinical implications. Nat. Rev. Clin. Oncol..

[10] Boj S.F., Hwang C.-I., Baker L.A., Chio I.I.C., Engle D.D., Corbo V., Jager M., Ponz-Sarvise M., Tiriac H., Spector M.S. (2015). Organoid models of human and mouse ductal pancreatic cancer. Cell.

[11] Huang L., Holtzinger A., Jagan I., BeGora M., Lohse I., Ngai N., Nostro C., Wang R., Muthuswamy L.B., Crawford H.C. (2015). Ductal pancreatic cancer modeling and drug screening using human pluripotent stem cell–and patient-derived tumor organoids. Nat. Med..

[12] Driehuis E., van Hoeck A., Moore K., Kolders S., Francies H.E., Gulersonmez M.C., Stigter E.C., Burgering B., Geurts V., Gracanin A. (2019). Pancreatic cancer organoids recapitulate disease and allow personalized drug screening. Proc. Natl. Acad. Sci. USA.

[13] Gendoo D.M., Denroche R.E., Zhang A., Radulovich N., Jang G.H., Lemire M., Fischer S., Chadwick D., Lungu I.M., Ibrahimov E. (2019). Whole genomes define concordance of matched primary, xenograft, and organoid models of pancreas cancer. PLoS Comput. Biol..

[14] Tiriac H., Belleau P., Engle D.D., Plenker D., Deschênes A., Somerville T.D., Froeling F.E., Burkhart R.A., Denroche R.E., Jang G.-H. (2018). Organoid Profiling Identifies Common Responders to Chemotherapy in Pancreatic CancerPancreatic Cancer Organoids Parallel Patient Response. Cancer Discov..

[15] Aguirre A.J., Nowak J.A., Camarda N.D., Moffitt R.A., Ghazani A.A., Hazar-Rethinam M., Raghavan S., Kim J., Brais L.K., Ragon D. (2018). Real-time Genomic Characterization of Advanced Pancreatic Cancer to Enable Precision MedicineGenomic Precision Medicine in Advanced Pancreatic Cancer. Cancer Discov..

[16] van de Wetering M., Francies H.E., Francis J.M., Bounova G., Iorio F., Pronk A., van Houdt W., van Gorp J., Taylor-Weiner A., Kester L. (2015). Prospective derivation of a living organoid biobank of colorectal cancer patients. Cell.

[17] Collins A., Miles G.J., Wood J., MacFarlane M., Pritchard C., Moss E. (2020). Patient-derived explants, xenografts and organoids: 3-dimensional patient-relevant pre-clinical models in endometrial cancer. Gynecol. Oncol..

[18] Huo K.-G., D’Arcangelo E., Tsao M.-S. (2020). Patient-derived cell line, xenograft and organoid models in lung cancer therapy. Transl. Lung Cancer Res..

[19] Inoue A., Deem A.K., Kopetz S., Heffernan T.P., Draetta G.F., Carugo A. (2019). Current and future horizons of patient-derived xenograft models in colorectal cancer translational research. Cancers.

[20] Xue X., Shah Y.M. (2013). In vitro organoid culture of primary mouse colon tumors. JoVE.

[21] Weeber F., van de Wetering M., Hoogstraat M., Dijkstra K.K., Krijgsman O., Kuilman T., Gadellaa-van Hooijdonk C.G., van der Velden D.L., Peeper D.S., Cuppen E.P. (2015). Preserved genetic diversity in organoids cultured from biopsies of human colorectal cancer metastases. Proc. Natl. Acad. Sci. USA.

[22] Roper J., Tammela T., Cetinbas N.M., Akkad A., Roghanian A., Rickelt S., Almeqdadi M., Wu K., Oberli M.A., Sánchez-Rivera F.J. (2017). In vivo genome editing and organoid transplantation models of colorectal cancer and metastasis. Nat. Biotechnol..

[23] Nagathihalli N.S., Castellanos J.A., Shi C., Beesetty Y., Reyzer M.L., Caprioli R., Chen X., Walsh A.J., Skala M.C., Moses H.L. (2015). Signal transducer and activator of transcription 3, mediated remodeling of the tumor microenvironment results in enhanced tumor drug delivery in a mouse model of pancreatic cancer. Gastroenterology.

[24] Shah A.T., Diggins K.E., Walsh A.J., Irish J.M., Skala M.C. (2015). In vivo autofluorescence imaging of tumor heterogeneity in response to treatment. Neoplasia.

[25] Walsh A.J., Cook R.S., Sanders M.E., Aurisicchio L., Ciliberto G., Arteaga C.L., Skala M.C. (2014). Quantitative optical imaging of primary tumor organoid metabolism predicts drug response in breast cancer. Cancer Res..

[26] Foley T.M., Payne S.N., Pasch C.A., Yueh A.E., Van De Hey D.R., Korkos D.P., Clipson L., Maher M.E., Matkowskyj K.A., Newton M.A. (2017). Dual PI3K/mTOR inhibition in colorectal cancers with APC and PIK3CA mutations. Mol. Cancer Res..

[27] Monberg M.E., Geiger H., Lee J.J., Sharma R., Semaan A., Bernard V., Wong J., Wang F., Liang S., Swartzlander D.B. (2022). Occult polyclonality of preclinical pancreatic cancer models drives in vitro evolution. Nat. Commun..

[28] Sharick J.T., Walsh C.M., Sprackling C.M., Pasch C.A., Pham D.L., Esbona K., Choudhary A., Garcia-Valera R., Burkard M.E., McGregor S.M. (2020). Metabolic heterogeneity in patient tumor-derived organoids by primary site and drug treatment. Front. Oncol..

[29] Larsen B.M., Kannan M., Langer L.F., Leibowitz B.D., Bentaieb A., Cancino A., Dolgalev I., Drummond B.E., Dry J.R., Ho C.-S. (2021). A pan-cancer organoid platform for precision medicine. Cell Rep..

[30] Sakamoto H., Attiyeh M.A., Gerold J.M., Makohon-Moore A.P., Hayashi A., Hong J., Kappagantula R., Zhang L., Melchor J.P., Reiter J.G. (2020). The Evolutionary Origins of Recurrent Pancreatic CancerOrigins of Recurrent Pancreatic Cancer. Cancer Discov..

[31] Juiz N.A., Iovanna J., Dusetti N. (2019). Pancreatic cancer heterogeneity can be explained beyond the genome. Front. Oncol..

[32] Lomberk G., Blum Y., Nicolle R., Nair A., Gaonkar K.S., Marisa L., Mathison A., Sun Z., Yan H., Elarouci N. (2018). Distinct epigenetic landscapes underlie the pathobiology of pancreatic cancer subtypes. Nat. Commun..

[33] Miao Y., Ha A., de Lau W., Yuki K., Santos A.J., You C., Geurts M.H., Puschhof J., Pleguezuelos-Manzano C., Peng W.C. (2020). Next-generation surrogate Wnts support organoid growth and deconvolute frizzled pleiotropy in vivo. Cell Stem Cell.

[34] Joulia J., Pinguet F., Ychou M., Duffour J., Astre C., Bressolle F. (1999). Plasma and salivary pharmacokinetics of 5-fluorouracil (5-FU) in patients with metastatic colorectal cancer receiving 5-FU bolus plus continuous infusion with high-dose folinic acid. Eur. J. Cancer.

[35] Graham M.A., Lockwood G.F., Greenslade D., Brienza S., Bayssas M., Gamelin E. (2000). Clinical pharmacokinetics of oxaliplatin: A critical review. Clin. Cancer Res..

[36] Chabot G.G. (1997). Clinical pharmacokinetics of irinotecan. Clin. Pharmacokinet..

[37] Kroep J.R., Giaccone G., Voorn D.A., Smit E.F., Beijnen J.H., Rosing H., van Moorsel C.J., van Groeningen C.J., Postmus P.E., Pinedo H.M. (1999). Gemcitabine and paclitaxel: Pharmacokinetic and pharmacodynamic interactions in patients with non–small-cell lung cancer. J. Clin. Oncol..

[38] Ueno H., Ikeda M., Ueno M., Mizuno N., Ioka T., Omuro Y., Nakajima T.E., Furuse J. (2016). Phase I/II study of nab-paclitaxel plus gemcitabine for chemotherapy-naive Japanese patients with metastatic pancreatic cancer. Cancer Chemother. Pharmacol..

[39] Noble S., Goa K.L. (1997). Gemcitabine. Drugs.

[40] Pasch C.A., Favreau P.F., Yueh A.E., Babiarz C.P., Gillette A.A., Sharick J.T., Karim M.R., Nickel K.P., DeZeeuw A.K., Sprackling C.M. (2019). Patient-derived cancer organoid cultures to predict sensitivity to chemotherapy and radiation. Clin. Cancer Res..

[41] Kratz J.D., Rehman S., Johnson K.A., Gillette A.A., Sunil A., Favreau P.F., Pasch C.A., Miller D., Zarling L.C., Yeung A.H. (2021). Integrating Subclonal Response Heterogeneity to Define Cancer Organoid Therapeutic Sensitivity. bioRxiv.

[42] Aisenbrey E.A., Murphy W.L. (2020). Synthetic alternatives to Matrigel. Nat. Rev. Mater..

[43] LeSavage B.L., Suhar R.A., Broguiere N., Lutolf M.P., Heilshorn S.C. (2022). Next-generation cancer organoids. Nat. Mater..

[44] Osuna de la Peña D., Trabulo S.M.D., Collin E., Liu Y., Sharma S., Tatari M., Behrens D., Erkan M., Lawlor R.T., Scarpa A. (2021). Bioengineered 3D models of human pancreatic cancer recapitulate in vivo tumour biology. Nat. Commun..

[45] Hirokawa Y., Clarke J., Palmieri M., Tan T., Mouradov D., Li S., Lin C., Li F., Luo H., Wu K. (2021). Low-viscosity matrix suspension culture enables scalable analysis of patient-derived organoids and tumoroids from the large intestine. Commun. Biol..

[46] DeStefanis R.A., Kratz J.D., Olson A.M., Sunil A., DeZeeuw A.K., Gillette A.A., Sha G.C., Johnson K.A., Pasch C.A., Clipson L. (2022). Impact of baseline culture conditions of cancer organoids when determining therapeutic response and tumor heterogeneity. Sci. Rep..

