# Supplementary material for: Spatial Alignment of Organoids Tracking Subclonal Chemotherapy Resistance in Pancreatic and Ampullary Cancer
Source: Bioengineering (Basel). 2023 Jan 10;10(1):91. doi: 10.3390/bioengineering10010091 (PMC9854538; doi:10.3390/bioengineering10010091)
Supplement: Supplementary file 1 [file bioengineering-10-00091-s001.zip › bioengineering-2133328-supplementary.pdf]

Supplementary Materials

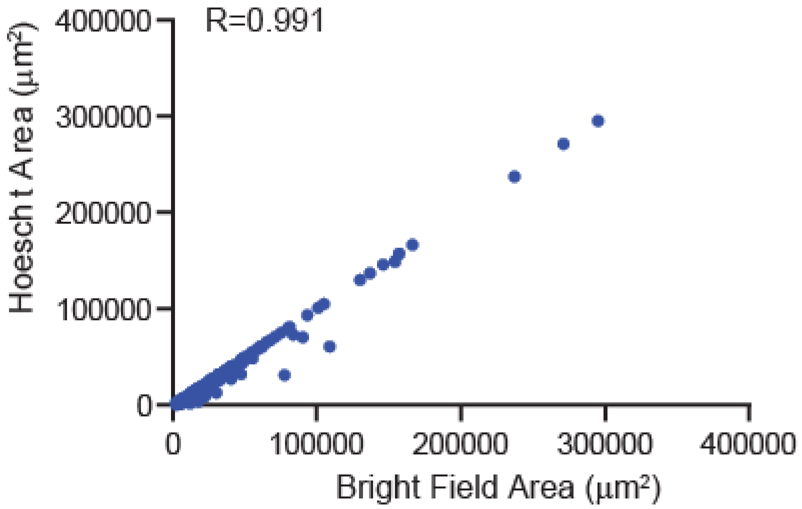

Figure S1. Comparison of individual organoid size. Thresholded images compared between brightfield area versus Hoescht staining (n=276).

Table S1. Summary of media composition used for pancreatic and ampullary PCOs.

Name	Component	Supplier & Catalog	Final Concentration	Application
Base Media	Advanced DMEM/F12	Gibco 12634-010	1x	Washing, Passaging
	Glutamax, 100x	Gibco 35050-061	1x	
	Penicillin/Streptomycin	Lonza 17-602E	100 U/mL-100 ug/mL	
Synthetic Feeding Media	Base Media	(as above)	100% v/v	Culture Propagation
	HEPES, 1M	Gibco 15630-080	10mM	
	B27 Supplement, 50x	Gibco 17504-001	0.4x	
	Nicotinamide	Sigma N0636-100G	10mM	
	N-Acetyl-L-Cysteine	Sigma A9165-25G	0.5mM	
	A83-01	Sigma SML0788-5MG	500nM	
	SB202190	MCE HY-10295	500nM	
	Wnt Surrogate Fusion Peptide	ImmunoPrecise N001-1mg	500pM	
	Human Recombinant Noggin	Acro NON-H5257	100ng/mL	
	HA-R-Spondin 1	(in house)	500pM	
	Y-27632	MCE HY-10071	10nM	
	Human Recombinant EGF	Gibco PHG0313	50ng/mL	

Table S2. Summary of clinical parameters for individual organoid cultures.

Line	Primary	Site for Organoids	Histology	Grade	AJCC Staging	Recurrence
AMPC1	Ampullary	Primary	Adenocarcinoma	Moderate	pT2 pN1 M0	No
PANC1	Pancreatic	Metastatic (pleural effusion)	Ductal Adenocarcinoma	Moderate	cT3 cN1 M0	Yes
PANC2	Pancreatic	Metastatic (liver core)	Ductal Adenocarcinoma	Moderate to poor	cT3 cN2 M1	n/a
